# Drop the Needle; A Temperature Stable Oral Tablet Vaccine Is Protective against Respiratory Viral Pathogens

**DOI:** 10.3390/vaccines10040593

**Published:** 2022-04-12

**Authors:** Becca A. Flitter, Molly R. Braun, Sean N. Tucker

**Affiliations:** Vaxart, Inc., South San Francisco, CA 94080, USA; mbraun@vaxart.com (M.R.B.); stucker@vaxart.com (S.N.T.)

**Keywords:** oral vaccines, mucosal immune response, adenoviral vector vaccine, IgA, vaccine tablet, antibody-secreting cells

## Abstract

To effectively combat emerging infections and prevent future pandemics, next generation vaccines must be developed quickly, manufactured rapidly, and most critically, administered easily. Next generation vaccines need innovative approaches that prevent infection, severe disease, and reduce community transmission of respiratory pathogens such as influenza and SARS-CoV-2. Here we review an oral vaccine tablet that can be manufactured and released in less than 16 weeks of antigen design and deployed without the need for cold chain. The oral Ad5 modular vaccine platform utilizes a non-replicating adenoviral vector (rAd5) containing a novel molecular TLR3 adjuvant that is delivered by tablet, not by needle. This enterically coated, room temperature-stable vaccine tablet elicits robust antigen-specific IgA in the gastrointestinal and respiratory tracts and upregulates mucosal homing adhesion molecules on circulating B and T cells. Several influenza antigens have been tested using this novel vaccine approach and demonstrated efficacy in both preclinical animal models and in phase I/II clinical trials, including in a human challenge study. This oral rAd5 vaccine platform technology offers a promising new avenue for aiding in rapid pandemic preparedness and equitable worldwide vaccine distribution.

## 1. Introduction

The recent pandemic caused by severe acute respiratory syndrome coronavirus-2 (SARS-CoV-2) demonstrated that public health systems throughout the world were inadequately prepared to respond to an outbreak of a novel pathogen. Although innovative vaccines were quickly developed and manufactured in record time, the storage requirements and slow distribution continue to be a significant obstacle to achieving rapid vaccine rollout [1]. Currently, SARS-CoV-2 vaccines in use around the world require cold chain storage and robust infrastructure that mobilizes healthcare providers to administer vaccines. Many developing countries lack the public health resources and infrastructure to quickly implement new strategies in response to emergent infections, posing challenges to equitable healthcare delivery [2,3]. To rapidly respond to future pandemics in a manner that ensures global uptake and equitable health outcomes, new vaccine technologies must be simple to administer and ideally maintain potency for long periods of time at room temperature [4].

In addition to the challenges posed by vaccine storage and distribution, vaccine hesitancy within industrialized nations, especially regarding injections, impedes adequate immunization rates. A recent poll conducted by Quadrant Strategies reveals that a phobia of injections is a deterrent to those deciding whether to become immunized [5]. The poll showed that 32% of individuals who didn’t plan on obtaining a SARS-CoV-2 vaccine indicated they would be more likely to opt for pill option rather than a parenteral immunization. Furthermore, 70% of those polled would prefer taking a pill on their own schedule rather than making an appointment with a provider, which can be time consuming and inconvenient and may require time off work. This highlights the fact that a self-administered tablet vaccine could lead to better vaccination coverage.

A room temperature-stable tablet has been developed that utilizes non-replicating adenoviral vector technology and a molecular toll-like receptor 3 (TLR3) adjuvant. This tablet vaccine platform can be employed to target a variety of pathogenic viruses and has been tested in several phase I/II clinical trials, including those for influenza, SARS-CoV-2, and norovirus [6,7,8,9,10]. In this review, we will discuss how this oral tablet vaccine elicits antigen-specific systemic and mucosal responses against influenza and SARS-CoV-2 in both clinical and pre-clinical studies.

## 2. Developing Next Generation Vaccines

Since the first inactivated influenza vaccines were developed in the 1940s, few improvements in technologies or manufacturing processes have been adopted. Until 2009, the overwhelming majority of influenza vaccines were produced in eggs, a method that takes a minimum of 6 months [11]. New technologies have been developed for influenza vaccine development, including cell culture and recombinant protein-based approaches. However, of the six commercially available vaccines distributed in the United States for the 2021–2022 season, four still utilized egg-based production [12]. Due to the long lead time required for egg-based vaccine production, recommendations for candidate vaccine antigens must be made 6–8 months prior to rollout. The Global Influenza Surveillance and Response System (GISRS), a team of 120 national laboratories, tracks influenza subtypes and antigenic drift in the Northern and Southern hemispheres and makes recommendations on vaccine antigens that might match the following year’s dominant circulating influenza strain [13]. Unfortunately, accurately selecting vaccine strains to include each year over six months in advance is challenging. A recent example was the antigen drift of influenza A strain H3N2 during the 2014–2015 flu season, resulting in a vaccine efficacy of less than 19% [14]. Antigen mismatch, egg-based manufacturing constraints, and the time required to administer parenteral vaccines may not adequately protect the population in a timely manner against novel flu strains. Inactivated flu vaccines are modified to be efficiently propagated in chicken eggs, which may inadvertently lead to significant antigenic changes [15]. In the 2016–2017 season, the loss of a single glycosylation site stemming from an egg-adapted mutation of the virus strain may have had profound effects on vaccine efficacy, as antibodies generated against the modified hemagglutinin were inefficient at neutralizing circulating viruses [16]. Additionally, new vaccines for respiratory pathogens must be able to quickly adapt, as RNA viruses can mutate due to genetic variability and antigenic drift [17]. Therefore, newer strategies are needed, such as cell-based manufacturing or recombinant DNA technology that both reduce the long lead time and lower the risk of vaccine antigen mismatch [15].

Recently, technologies that greatly reduce vaccine manufacturing time have been granted Emergency Use Authorization by the Food and Drug Administration. As demonstrated in the recent COVID-19 pandemic, mRNA and non-replicating recombinant adenoviral (rAd) vector vaccines can be produced much faster than traditional methods and are effective at producing high levels of serum antibodies [18,19,20]. However, cold-chain requirements, especially for the mRNA vaccines, presented a major obstacle for transport, storage, and distribution during the SARS-CoV-2 pandemic. Temperature requirements for these new technologies have resulted in global vaccine distribution inequity and have become a large hurdle for countries that lack resources to properly implement the necessary storage infrastructure. While manufacturing processes for newer vaccine technologies have improved production speed, the continual requirements for trained personnel and adequate infrastructure to administer parenteral vaccines is a major barrier for rapid distribution during a pandemic. Advances in vaccine technology have been recently implemented in developed nations, and low-income countries have been left behind, amplifying healthcare disparities and emphasizing the urgent need for improvements in vaccine storage and global distribution [21,22].

**Figure 1:**
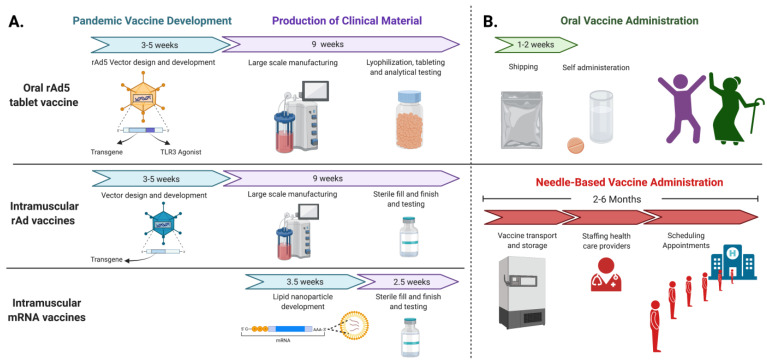
Next generation vaccine production, manufacturing, and distribution timelines during a pandemic. (**A**) Non-replicating recombinant adenovirus serotype 5 (rAd5) vaccine constructs can be developed and used in preclinical testing in as little as three weeks. Large scale manufacturing and release timelines for rAd-based vaccines are comparable and can be completed within 9 weeks, as demonstrated during the 2020 pandemic (S. Gilbert, personal communication, 28 October 2021). mRNA-based vaccines have the shortest development and manufacturing timelines; however, these formulations are the most difficult to transport and store [18,23]. (**B**) An oral tablet rAd5 vaccine that is room temperature-stable can be shipped without cold chain and does not need to be administered by trained health care professionals. Distribution of an oral vaccine can exponentially speed up vaccine rollout compared to injected vaccines, especially in areas with considerably fewer resources, as tablets can be shipped directly to individuals and self-administered. Traditional needle-based immunization approaches have an inherent distribution bottleneck due to temperature constraints for shipping and storage and the need for health care professionals to administer the vaccines. Administering needle-based vaccines seems to be the greatest bottleneck and can take more than 6 months, even with substantial investment in infrastructure.

An oral rAd tablet vaccine that can be rapidly produced and deployed, is suited to transforming global vaccination strategies by removing cold-chain requirements and the necessity of administration by healthcare professionals. rAd-based vaccine approaches are also modular and can be easily modified to carry different vaccine antigens. The flexibility of this technology allows for the rapid development of new vaccines following the emergence of unique influenza virus strains or novel pathogens. Another benefit of rAd vector vaccines is their quick production timeline by cell culture manufacturing techniques, which allows for the generation of clinical material in less than 9 weeks (Figure 1A). While mRNA formulations can be manufactured rapidly and currently have the shortest time to market, their inherent instability at temperatures above −70 °C results in numerous discarded doses [23]. It is estimated that about a million SARS-CoV-2 vaccine doses were wasted in the United States between December 2020 and July 2021 due to temperature deviations [24]. This limitation does not apply to an oral vaccine tablet, which is designed to be room temperature-stable for multiple years. Furthermore, injected vaccines generate excessive biohazardous waste, such as syringes and sanitation materials during vaccine campaigns [25], which can be greatly decreased by employing a tablet-based system. Most importantly, an oral tablet approach reduces the need for skilled practitioners to administer the vaccine, as it can be easily distributed and self-administered (Figure 1B). This fundamental change in delivery can accelerate vaccine rollout during pandemics by eliminating cold storage and infrastructure requirements while also reducing biowaste and unused vaccine doses. Lastly, another advantage of rAd oral tablet administration is the adverse events experienced after vaccination appear to be quite mild, without any signs of injection site pain or fever when compared to a placebo group [8]. That is not the case with the current mRNA vaccines or injected adenovirus vaccines, where fever, headache and chills are quite common outcomes post immunization [26,27,28,29].

Room temperature-stable vaccines could also be beneficial for stockpiling vaccine supply, allowing for rapid distribution during a pandemic. The potency of the oral rAd5 tableted vaccine drug product has been evaluated at multiple temperatures over extended storage times. Vaccine drug products for human use must be manufactured under regulations known as good manufacturing practices (GMP). In preparation for a phase II clinical influenza challenge study, a GMP-manufactured monovalent hemagglutinin vaccine drug product (VXA-A1.1) was placed at +25 °C at 60% relative humidity for a period exceeding two years. Tablets used in the influenza challenge study retained potency, as measured by viral infectivity assay, which showed that the drug product remained within all tested specifications for up to 426 days (Table 1). GMP tablets used in norovirus clinical trials, have also been extensively tested and remained within specification for 246 days at 30 °C and can tolerate higher temperatures (Table 1). These results demonstrate that an oral rAd5 vaccine tablet is notably stable at room temperature and could simplify vaccine distribution to lower-resource areas where cold chain transport is unavailable.

## 3. Advantages of Orally Delivered rAd Vaccines

Non-replicating rAd vectors are attractive vaccine delivery platforms due to their effective antigen expression and presentation, which elicit innate and adaptive cellular and humoral immune responses [30]. When rAd vaccines are delivered to the host, target antigens are expressed intracellularly and can be displayed on the cell surface or secreted, depending on the targeting sequence. Intracellular vaccine antigen expression also leads to constitutive processing and the presentation of peptides through MHC-class I and II, like natural infection, provoking robust T cell responses [31,32]. Mimicking the natural protein conformation of vaccine antigens, rAd vectors elicit broadly protective antibodies that are not easily produced from injected protein or subunit vaccines, where the protein can refold during manufacturing to exist in the lowest energy state [33]. Several influenza-based studies demonstrate that native stable HA homotrimer conformation enhances immunogenicity and elicits broadly protective antibody responses [34,35]. However, intramuscular parenteral delivery of Ad vectors does not imitate the natural infection of respiratory pathogens and therefore does not elicit mucosal responses. The high number of breakthrough cases of SARS-CoV-2 following parenteral vaccine administration demonstrates the benefits of enhancing mucosal immune responses to reduce viral transmission [36].

The rAd vaccines that can be delivered to the mucosa are ideal for displaying vaccine antigens in a manner similar to what the immune system encounters during infection. However, oral delivery of Ad vectors in humans has been challenging to develop due to oral tolerance mechanisms that hinder immunogenicity [37], a major obstacle in the development of orally based vaccines [38,39]. The oral rAd5 vaccine platform uses a molecular TLR3 adjuvant to overcome oral tolerance in the small intestine (reviewed by [39]). TLR3 is a pattern recognition receptor that senses dsRNA, which can be made by many pathogenic viruses during intracellular replication [40]. In the human small intestine, TLR3 protein is highly expressed by enterocytes, primarily in the villi [41,42,43]. When TLR3 is activated, inflammatory cascades and anti-viral responses are elicited, producing the release of both IFN types I and III. These potent, soluble, anti-viral mediators have been shown to enhance plasmablast differentiation and T cell activation [44,45,46]. Preclinical murine models have demonstrated synthetic double-stranded RNA as an effective adjuvant when administered intranasally in conjunction with an inactivated split HA influenza vaccine [47]. This study also demonstrated that IgA secretion in nasal wash and systemic IgG responses were both enhanced by mucosal delivery of TLR3 and vaccine antigens. Tissue-resident dendritic cells also respond to TLR3 stimulation and have been shown to be key in inducing influenza mucosal vaccine-specific responses including CD8 T cells [48]. This approach, tested in a preclinical model, demonstrated the administration of intranasal polyinosine-polycytidylic (polyI:C) in conjunction with an influenza subunit vaccine, enhanced antigen-specific IgA responses [48]. By including a molecular TLR3 adjuvant that is expressed by the viral vector, this vaccine technology has overcome a major barrier for effective oral rAd delivery.

## 4. Mucosal Vaccination Elicits Cellular and Humoral Responses

The generation of pathogen-specific antibodies in the serum that are capable of neutralizing pathogens is one of the main hallmarks of measuring vaccine-induced immunogenicity. Circulating B cells that produce antibodies are known as antibody-secreting cells (ASCs) and can differentiate into plasmablasts that can traffic to immune sites, such as bone marrow, lymph nodes, and the spleen. In mucosal tissues, plasmablasts predominantly secrete dimeric IgA into the lumen, and due to increased valency, this immunoglobulin has been shown to have higher cross-reactive neutralizing activity against invading pathogens and viral variants than IgG [49,50]. Lymphocytes express various integrins that assist circulating immune cells in homing to various tissues in the body. The differential upregulation of surface integrins on circulating ASCs has been demonstrated to be important for lymphocyte homing [51]. Mucosal homing B and T cells express the integrin α4β7+, which recognizes the mucosal addressin cellular adhesion molecule-1 (MAdCAM-1), highly expressed on endothelial cells lining the mucosa [52,53]. A clinical study comparing vaccination administration routes found that mucosal delivery elicited α4β7+ ASCs, whereas injected preparations upregulated the lymph node homing marker L-selectin (CD62L+) [53]. Following exposure to vaccine antigens in the small intestine, lamina propria resident dendritic cells and epithelial cells can cross present antigens to naïve T cells through MHC I and II [31,32]. Activated T cells migrate to intestinal lymphoid tissue where T follicular helper cells (Tfh) support the B cell maturation and ASC development. Lastly, studies have shown that immune signals from the gut mucosa can support immune responses in the respiratory tract, providing evidence that there is cross talk between distal mucosal sites [38,54]. Therefore, mucosal immunization can offer a significant protective benefit over injected vaccines by enhancing IgA production and cellular immune responses at the principal sites of infection.

**Figure 2:**
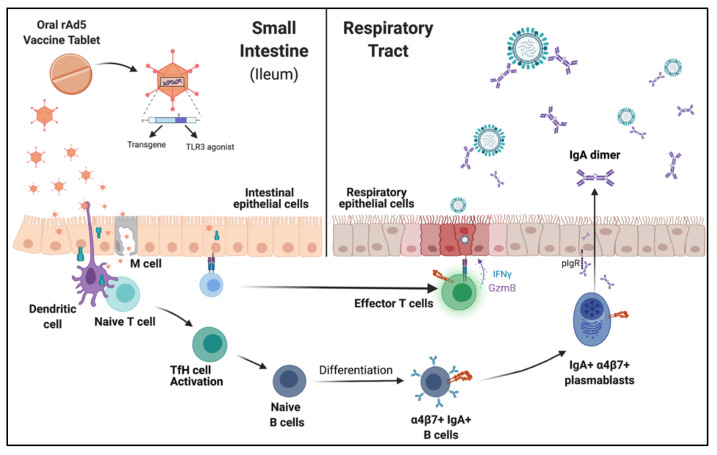
Working model of oral rAd5 vaccine tablet, illustrating how antigen delivery to the small intestine generates protective mucosal immune responses. The non-replicating rAd5 vector contains both the vaccine antigen transgene and the molecular TLR3 adjuvant, which are delivered together to epithelial and resident immune cells. Once released in the gut ileum, the tablet’s enteric coating dissolves and releases rAd5 into the lumen. Following translation of the transgene, the vaccine protein antigen can be displayed on target cells in native confirmation or cross-presented to T cells as peptides on MHC I and II. Constantly sampling the intestinal lumen are other antigen-presenting immune cells, such as dendritic cells (DCs), that can display non-self antigens to naïve T cells and drive T follicular (TfH) expansion. TfH cells provide co-stimulation that enhances B cell differentiation, maturation, and class-switch recombination to IgA-expressing activated B cells. Activated B cells mature into IgA-secreting plasmablasts and enter the lymph and blood, where they traffic to lymphoid tissues and mucosal sites. Following oral vaccination, circulating IgA antibody-secreting cells upregulate the mucosal homing marker α4β7+, traffic to the respiratory tract, and secrete IgA. IgA transcytosis through the epithelial layer is mediated by the polymeric Ig receptor (pIgR) and released into the airway. Resident T cells may also recognize vaccine antigens displayed on the surface of gut epithelial cells or dendritic cells and elicit effector T cell maturation. These effector T cells can provide additional mucosal protection by identifying naturally infected cells and releasing effector molecules, such as IFNγ and granzyme (GzmB), in response to infection.

Oral administration of influenza antigens using the rAd5 tablet vaccine elicits both humoral and cellular mucosal pathogen-specific responses, leading to a working model base in experimental and clinical studies (Figure 2). A single dose of oral rAd5 vaccine tablet containing the influenza HA antigen, when delivered specifically to the ileum by radio-controlled capsule, elicited strong antigen-specific systemic and mucosal responses [6]. Immunized subjects showed an increase in α4β7+, IgA+ B cells and increased HA-specific IgG- and IgA-specific ASC’s [6]. This demonstrated that oral vaccination by a non-replicating vector can shift ASC immunophenotyping profiles and enhance plasmablast homing to mucosal sites. Furthermore, HA-specific IgA in fecal and nasal samples was elevated following vaccination, an observation that was mirrored by preclinical studies in ferret models of enteric vaccine delivery [55]. In a phase I study, subjects immunized with an oral rAd5 HA-expressing vaccine tablet elicited IgG and IgA ASC’s, which correlated with increases in hemagglutination inhibition and microneutralization assays [8]. Together, these data established that the rAd5-based oral vaccine platform elicits immunogenic mucosal humoral responses.

In addition to antibody-mediated immunity, antiviral responses by T cells have been shown to be important for protection against influenza [56], particularly in the elderly where immunosenescence may impact immune responses [57,58]. Oral vaccination using rAd5 increased the CD8+ and CD4+ T cell responses (memory and effector populations) post vaccination, as measured using a 40-antibody panel and mass-cytometry [9]. Multiple T cell subsets meaningfully contributed to protection by multivariate analysis [9]. Additionally, in a recent phase I clinical safety trial, individuals who received first generation oral SARS-CoV-2 rAd5 vaccine had elevated anti-viral circulating CD8+ T cells ([59] and manuscript in preparation). S-specific increases in IFNγ, CD107α, and TNFα were detected in CD8+ T cells by intracellular flow cytometry analysis following prime and boost immunizations 30 days apart. Furthermore, CD8+ T cell responses in individuals that received the oral rAd5 vaccine series were of higher magnitude than currently licensed vaccines when run in a comparator study [59]. Additional studies are currently underway to further understand T cell responses elicited by oral rAd5 vaccine tablets and how these potent cells contribute to vaccine-mediated protection.

## 5. Mucosal IgA and Influenza Vaccine Efficacy

Eliciting pathogen-specific mucosal immune responses is ideal for effective prevention of influenza and other microorganisms that enter the host through mucosal surfaces [60]. One of the hallmarks of mucosal responses is dimeric secretory IgA (SIgA), which plays an important role in prohibiting pathogen adherence and excluding infectious particles from gaining access to the host [61]. Due to its valency, SIgA has more potent neutralizing and cross-reactive activity than IgG and has been shown to provide enhanced protection against influenza antigenic drift [62,63,64]. This immunoglobulin is secreted at mucosal tissues including the nasopharynx, which is the first line of defense the host has for protection against influenza or other airborne pathogens. SIgA contains a J chain that binds the polymeric immunoglobulin receptor pIgR, allowing for transcytosis from the basolateral to the apical side of epithelial cells [65]. IgA also has intracellular neutralizing activity, another important host defense function at the mucosal surface [66,67,68]. There have been several vaccine candidates that elicit antigen-specific nasal IgA responses, which correlates with protection against influenza virus infection [69,70,71]. Additionally, methods have not yet been established for standardizing measurements for accurately quantifying mucosal IgA responses, making it challenging to implement in large clinical studies.

Most of the influenza vaccines currently on the market received licensure based on hemagglutination inhibition assays (HAI), which measure the functional activity of serum antibodies. While a serum HAI titer of greater than 40 is conventionally accepted as sufficient for protection against influenza [72,73], immunized individuals with high serum HAI are not always protected from disease [74]. Influenza virus diversity and antigenic drift among circulating strains impedes HAI as a predictive measurement for vaccine efficacy, as vaccine antigens do not always match circulating strains [75]. Furthermore, HAI is not always an accurate predictor of vaccine efficacy, especially when other routes of administration are used [74]. For example, the intranasal live attenuated influenza vaccines elicit considerable nasal IgA responses but less serum antibody [76]. Other humoral responses to influenza include non-neutralizing Fc-effector functions that induce antibody-mediated cellular cytotoxicity, and these also have been shown to be important for protection [77,78]. Therefore, solely utilizing HAI as the key metric for evaluating new mucosal vaccine technologies will continue to be a significant hurdle, as serum and blood collection remains the preferred method for tracking efficacy. Therefore, gauging influenza vaccine efficacy by only measuring systemic humoral responses does not accurately reflect all correlates of protection, especially for mucosal vaccine approaches.

## 6. Humoral Reponses in the Mucosa Are Protective against Challenge and Reduce Transmission

Parenteral vaccines are designed to induce systemic pathogen-specific IgG and memory responses and reduce severe disease. However, they do not induce antigen-specific mucosal responses, which may be necessary to reduce community-wide transmission during a pandemic. The ongoing SARS-CoV-2 pandemic has created a situation where parenterally immunized individuals, while protected from severe disease and death, are still able to become infected and transmit the virus to other people. A mucosal antibody response in the upper respiratory tract can block viral transmission more effectively than a vaccine that only elicits serum antibodies [76]. This observation has been demonstrated in preclinical models where dimeric IgA responses in the mucosa are more effective for reducing transmission than serum IgG responses. An influenza transmission study using a guinea pig model demonstrated that a recombinant neutralizing IgA antibody was highly effective at blocking transmission, but recombinant IgG with the same variable region administered to animals by injection was not effective [79]. Additionally, the recombinant IgG administered intranasally was able to block infection, indicating that the location of the neutralizing antibody is an important factor, not just the isotype. Therefore, strategically designing vaccines to elicit protective mucosal IgA responses may increase vaccine efficacy.

Mucosal delivery of vaccine antigens also has been demonstrated to be effective for reducing viral transmission to naïve animals. In a hamster study, animals mucosally immunized with rAd5-expressing SARS-CoV-2 spike protein and subsequently infected with SARS-CoV-2 virus had a significant reduction in their ability to transmit the virus to naïve animals via unidirectional aerosol airflow [10]. This study also demonstrated that vaccination routes of the same antigen resulted in significant differences in viral transmission. Viral copies measured in naïve animals’ nasal passages following exposure to mucosally immunized index animals was significantly reduced compared to naïve animals exposed to index animals vaccinated with the same vaccine by intramuscular injection. These studies illustrate that oral immunization with an oral rAd5 vaccine can elicit antigen-specific responses in the mucosa and reduce transmission to naïve animals.

## 7. Evaluating Novel Immune Correlates for Mucosal Vaccines

Correlates of protection for mucosal vaccines may likely be different for injected immunizations (reviewed by [38]). Most influenza vaccines are evaluated based on serum HAI titers; however, this presents an obstacle for oral formulations that are designed to generate immune responses at the mucosa. To determine the appropriate immune correlate to properly assess efficacy of the oral rAd5 influenza vaccine tablet, a human challenge study was conducted [7]. Prior to intranasal infection with the H1N1 virus, subjects were randomized to receive either a placebo, commercially available quadrivalent FluZone (IIV), or an oral rAd5 tablet H1N1 influenza vaccine (VXA-A1.1) [7]. Although both vaccines protected the majority of subjects from illness (Table 2), the group that received VXA-A1.1 had lower symptomatic disease compared to subjects immunized intramuscularly with FluZone (29%, 35% respectively). Furthermore, in a post-hoc analysis, viral shedding was decreased in subjects immunized with VXA-A1.1 compared to IIV (80% probability of superiority based on Bayesian analysis), demonstrating that administration of an oral rAd5 vaccine may further reduce viral shedding of the influenza virus compared to current licensed vaccines. Serum HAI responses were higher in the IIV-immunized cohort compared to the oral VXA-A1.1 group, indicating that serum antibody titers may not be the most important correlate for assessing the efficacy of the oral rAd5 vaccine. By utilizing a random forest analysis, a predictive machine learning algorithm that examines complex and diverse datasets as an ensemble, cellular responses measured by flow cytometry were found to be the most important correlate for VXA.A1-1 efficacy. More specifically, in subjects immunized with VXA.A1-1, circulating antigen-specific ASCs and α4β7+ mucosa homing lymphocytes were significantly elevated and correlated with a reduction in symptomatic disease. This study illustrates that only measuring serum-based responses is not sufficiently predictive of protective efficacy for mucosal vaccines.

Developing novel cellular immunogenicity assays that can be used in large scale clinical trials to measure efficacy is critical for the development of next-generation vaccines. Measuring antigen-specific cellular responses is challenging in clinical trials; however, more recent technological advances have been facilitating the development of new correlates in clinical trials. The use of fixed whole blood and other advanced screening techniques has further enhanced the identification and quantification of important cellular phenotypic markers that can be used to evaluate protective efficacy [9]. In a follow-up to the human influenza challenge phase II study, fixed whole blood and mass cytometry analysis were used to measure B and T cell activation 8 days after vaccination [9]. In the VXA.A1.1 vaccine cohort, elevated α4β7+, phospho-Stat5+, and HA+ specific B cells highly correlated with a reduction in illness. Furthermore, central and effector CD8+ T cells in VXA-A1.1 recipients expressed higher β7 integrin and displayed an activated phenotype compared to placebo and FluZone recipients on day 8. Lastly, individuals in the VXA-A1.1 cohort who shed lower levels of the virus had elevated β7 integrin and pSTAT5 and low CD62L, indicating a correlation between mucosal homing and reduced viral load. This study illustrates that oral rAd5 vaccine administration induces protective levels of mucosal homing lymphocytes, which correlates with decreased viral shedding.

In addition to β7, α 4 can also dimerize with integrin β 1, and α 4 β 1+ B cells have been shown to migrate to various mucosal sites, including the bronchus-associated lymphoid tissue [51]. Both homing markers β 1 and β7 have been found in human peripheral blood ASCs following rAd5 oral vaccination, demonstrating that this vaccine technology induces a combination of intestinal and non-intestinal homing B cells [6,9]. In summary, to accurately measure mucosal vaccine efficacy, new cellular immunogenicity measurements need to be further developed for implementation in large clinical studies.

## 8. Anti-Vector Responses

One concern regarding the use of Ad vector vaccine platforms is the high rate of natural adenovirus seroprevalence and pre-existing immunity, which may impede efficacy. Several preclinical and clinical experiments were conducted to test if oral delivery of rAd5 to the small intestine generates anti-vector responses that interfere with vaccine antigen immunogenicity. In a ferret influenza challenge model utilizing H5N1, neutralizing antibody titers to rAd5 were quantified following a prime/boost regimen administered by injection or orally four weeks apart. The animals that received the vaccine via intramuscular administration developed substantial anti-Ad neutralizing antibodies. However, those animals vaccinated by oral administration with the same vector did not induce detectable anti-Ad5 responses [55]. Furthermore, preexisting immunity in humans to oral rAd5 had no effect on the ability to elicit neutralizing antibody responses or T cell responses following oral rAd5 immunization expressing influenza HA as an antigen nor did it have an effect on specific serum antibody responses in norovirus clinical trials [8,80]. Therefore, anti-vector responses that impede immunogenicity in injected rAd-based vaccines have less of a role when immunizing with oral rAd5.

## 9. Conclusions

The human mucosa interacts with numerous environmental and microbial agents every day, and as such, it is the first line of defense against respiratory pathogens, such as influenza and SARS-CoV-2. An oral rAd5 tablet vaccine described in this review has several advantages compared to traditional needle-based immunization approaches, including eliciting immune protective responses at primary sites of infection. Multiple clinical and pre-clinical studies have demonstrated that immunization with oral rAd5 elicits ASCs and T cells that express mucosal homing integrins and enhances pathogen-specific IgA responses. The combination of mucosal-directed humoral and cellular responses provides enhanced protection to respiratory pathogens, as shown by an influenza human challenge study. Recent studies have established that correlates of protection for an oral rAd5 mucosal vaccine are unique compared to currently licensed parenteral vaccines. Therefore, multiple serum, cellular, and mucosal endpoints will be evaluated in upcoming clinical trials to demonstrate immunogenicity and their correlation to efficacy.

While the systemic administration of vaccines via needle-based delivery has been successful for developing long-lasting immunity to many human pathogens, an oral rAd5 tablet-based vaccine platform has distinct advantages over the current parenteral vaccines, as it can be easily administered, stored at room temperature, and rapidly produced. An oral rAd5 vaccine has proven to be well-tolerated, safe, and immunogenic while also having a distinct distribution advantage as a room temperature-stable tablet that can be shipped globally and self-administered. Currently, countries with underdeveloped healthcare infrastructure are struggling to manage the cold-chain requirements, dosing schedules, and trained personnel required for the successful distribution and administration of COVID-19 vaccinations. The implementation of an oral rAd5 room temperature-stable, tablet-based vaccine would be a revolutionary tool that has the potential to make a significant impact on reducing the global burden of pathogenic viral diseases.

## Figures and Tables

**Table 1:** Oral rAd5 vaccine tablet stability.

Indication	Vaccine	Storage Temperature (°C)	Drug Product Within Specification
Influenza ^1^	VXA.A1.1	+25	426 days
Norovirus ^2^	VXA-G2.4-NS	+30	246 days
Norovirus ^2^	VXA-G2.4-NS	+40	34 days

^1^ Clinical Trial NCT03897309 ^2^ Clinical Trial NCT03897309.

**Table 2:** Vaccine efficacy following H1N1 challenge.

Study Arm	Number of Subjects	Viral Shedding ^1^	Illness ^2^	Most Important Correlate of Protection
VXA-A1.1	58	21 (36%)	17 (29%)	ASC IgA
IIV	54	24 (44%)	19 (35%)	Serum HAI
Placebo	31	22 (71%)	15 (48%)	NA

^1^ Detectable viral shedding by qPCR in nasopharyngeal swab on any day after the first 36 h after challenge. ^2^ Illness was defined based on self-reported symptoms, along with laboratory-confirmed infection.
